# A Multisensing Setup for the Intelligent Tire Monitoring

**DOI:** 10.3390/s17030576

**Published:** 2017-03-12

**Authors:** Francesco Coppo, Gianluca Pepe, Nicola Roveri, Antonio Carcaterra

**Affiliations:** 1 Department of Mechanical and Aerospace Engineering, Sapienza University of Rome, 00184 Rome, Italy; francesco.coppo@uniroma1.it (F.C.); gianluca.pepe@uniroma1.it (G.P.); nicola.roveri@gmail.com (N.R.); 2 CNIT, Consorzio Nazionale Interuniversitario per le Telecomunicazioni, 43124 Parma, Italy

**Keywords:** intelligent tire, smart tire, tire grip, autonomous vehicle, car monitoring

## Abstract

The present paper offers the chance to experimentally measure, for the first time, the internal tire strain by optical fiber sensors during the tire rolling in real operating conditions. The phenomena that take place during the tire rolling are in fact far from being completely understood. Despite several models available in the technical literature, there is not a correspondently large set of experimental observations. The paper includes the detailed description of the new multi-sensing technology for an ongoing vehicle measurement, which the research group has developed in the context of the project OPTYRE. The experimental apparatus is mainly based on the use of optical fibers with embedded Fiber Bragg Gratings sensors for the acquisition of the circumferential tire strain. Other sensors are also installed on the tire, such as a phonic wheel, a uniaxial accelerometer, and a dynamic temperature sensor. The acquired information is used as input variables in dedicated algorithms that allow the identification of key parameters, such as the dynamic contact patch, instantaneous dissipation and instantaneous grip. The OPTYRE project brings a contribution into the field of experimental grip monitoring of wheeled vehicles, with implications both on passive and active safety characteristics of cars and motorbikes.

## 1. Introduction and State of the Art of “Smart Tires”

Vehicle safety has been one of the subject of great interest for research centers, safety boards and car makers for many years. At the beginning of the 1980s, first electronic control systems that involved the contact between the tire and the road were introduced; one of them was the ABS, Antilock Braking System (ABS). This active control system and the ones that followed were based on an indirect estimation of the vehicle dynamics variables, such as forces, friction and load transfer to evaluate the tire-road friction factor by minimizing skid.

In the last twenty years, research has moved towards a different approach [1,2,3,4,5,6,7,8,9,10,11,12], which is based on using newly conceived sensors [9,10] to pursue the measurement of some key variables involving the tire-road contact interface [13,14,15,16], such as pressure, temperature, contact-patch dimensions, or even better wheel load [17], acceleration, friction factor [18,19,20] or deformation [21,22]. These new hardware and related algorithms are capable of measuring new quantities and extract additional information with potential use for the enhancement of the performance of the vehicle control systems, also putting the basis for breakthrough in new control devices.

All these systems rely on new sensors embedded in the tire to form the so so-called intelligent or smart tire [23,24,25,26,27,28,29], a system capable of monitoring directly and continuously these key parameters. The measured data are transferred to a logic control unit to be processed. Mathematical models able to put a light on the tire-road interface phenomena are also of great importance in this context. 

The acquired data can be used to start an alert, or for monitoring and controlling the entire vehicle dynamics. In the last years, several different types of monitoring systems have been produced. For example, the TPMS (Tire Pressure Monitoring System), is the archetypal system of intelligent tire and one of the few technologies that has found commercial use. Many other systems, such as those based on strain measurements, or accelerations via optical or magnetic devices, show enormous potentialities, even if their industrial use meets some cost difficulties when involving large mass production. 

The key elements that can contribute to the success of a new technology in this field are related to: (i) a relative simplicity in the construction process, (ii) the availability of low invasive (small size) sensors, (iii) the chance of simple and effective sensors power supply, (iv) the need of a robust system for data transmission, and (v) the need of robust interpretative models for the acquired data.

Among the solutions that obtained a good visibility in the context of intelligent tires, two macro-categories are worth mentioning, the former related to a direct acceleration measurement [6,8,13], the latter related to strain measurements [21,25,26,27], both of inner tire points. Works of Audisio [30], Morinaga [31] and Hannah [14] are more focused on a widespread industrial application of smart tire and employ commercial sensors, while others [8,13,15,25,32] use prototypal devices to study the nature of the phenomena involving the tire rolling.

The Optyre technology [33,34,35,36], developed at the Department of Mechanical and Aerospace Engineering, Sapienza, University of Rome, has the focus to build-up a new smart tire for the real-time identification of the tire stress, of the residual grip and of the rolling resistance, mainly thanks to direct optical strain measurement. The present technology is based on the results of a new patent [36] and presents the following characteristics: (i) use of Fiber Bragg Grating-FBG sensors based strain measurements, with the twofold advantage: (ii) the energy supply to the sensor is conveyed directly via the light beam (that is no battery or electrical wires are needed), and (iii) the information is transmitted on the same light beam, intrinsically a wireless transmission (no need of any radio transmitter or receiver). 

Optyre is focused on the direct strain measurement, leading to some important advantages with respect to the acceleration based technologies. This choice is due to the difficulty in evaluating strain from an acceleration measurement that is an ill-conditioned inverse problem [37], as extensively discussed in [34].

This paper describes the new frontiers of the Optyre project, related to a new experimental setup showing the ability of the system to acquire very good quality data on the rolling tire, identifying specific features of the signal. The whole project is divided in three different sections:

The first section of the project, presented in this paper, describes the development of a measurement system that relies on optical sensing lines [35], for a reliable acquisition system for the continuous measurement of the tire strain. This optical sensing line is obtained installing an array of FBG sensors in the tire carcass, provided with a series of smart solutions for the signal transmission. Furthermore, the optical measurement chain has been integrated with a parallel acquisition system composed by suitable sensors to analyze the optical measurement data. 

The second section regards the development of new models for the identification of the tire-road contact conditions, among them: The Brush Rod Beam model [34] where the study is mainly focused on the analytical definition of the phenomena at the tire-road interface, and the Optyre model [33], which employs a simpler analytical model to provide qualitative information concerning the rolling tire also outside the contact patch. 

The last section consists in the use of the acquired data as an input for the control logic that governs the vehicle dynamics, which is the third aim of the project. 

The paper is organized as follows. In Section 2, a description of the measuring hardware involved in the Optyre project is provided. The experimental campaign, carried out to test the performances of the developed prototype and to validate the theoretical models, is described in Section 3 and Section 4. An overview of the technological applications of the Optyre project is presented in Section 5. Among all the existing technologies in this field, some relevant solutions, providing state of the art tools for analyzing the contact patch phenomena in a rolling tire, are considered and compared to Optyre in Section 6. Some conclusive remarks with future developments are outlined in Section 7.

## 2. Hardware Setup

The Optyre system, developed by Carcaterra et al. [33], is a multi-sensing device that consists in a tire with embedded FBG and other sensors for the real-time identification of key parameters, such as the residual grip and the rolling resistance [35]. 

### 2.1. The Optical Sensing Line

The aim of the project is to develop an experimental system able to measure the circumferential strain of a rolling tire, embedding FBG sensors within the tire carcass.

As a quick reminder, Fiber Bragg Grating (FBG) based sensors are made recreating a spatially recurring alternation of the glass refraction index in a single mode optical fiber. The grating turns the fiber into an optical filter: in fact, a specifically narrowband infrared light fired into the fiber is reflected to a spectrum analyzer, while the rest of the light is fully transmitted.

**Figure 1:**
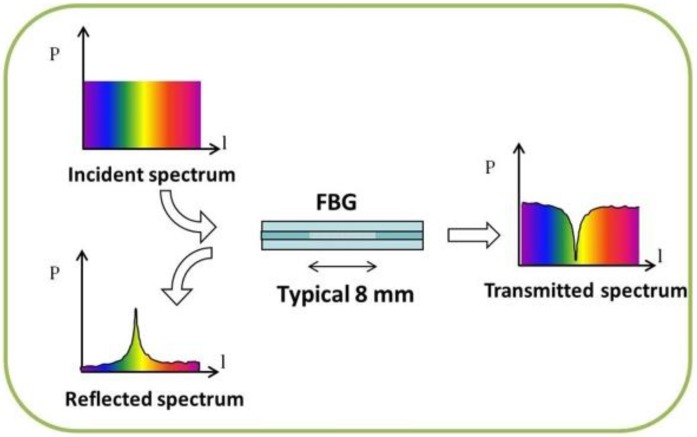
Light reflection diagram in a Fiber Bragg Grating-FBG sensor.

Observing the reflected spectrum, it is possible to recognize a peak value, called Bragg reflection wavelength (λ_B_), as shown in Figure 1. 

The value of the Bragg reflection wavelength depends on the effective refractive index, $n_{eff}$, and on the grating period, Λ, according to Equation (1), as presented by Antunes [38]:

(1) $$\lambda_{B}=2n_{\mathrm{eff}}\Lambda$$

when the FBG sensor is stretched, or compressed, $\lambda_{B}$ changes to λ, therefore, if the sensor is integrated with the point to measure, for example welding or pasting it to the monitored structure, the reflected wavelength changes are related to the strain and to the thermal variation, as described in Equation (2):

(2) $$\frac{\Delta \lambda }{\lambda_{B}}=\left(1-\rho_{e}\right)\varepsilon +\left(\alpha +\eta \right)\Delta T$$

where $\Delta \lambda =\lambda -\lambda_{B}$, $\rho_{e}$ represents the photo-elastic coefficient, ε is the mechanical strain, α is the thermal expansion of the silica, η represents the temperature dependence of the refractive index, and ΔT is the temperature variation.

**Figure 2:**
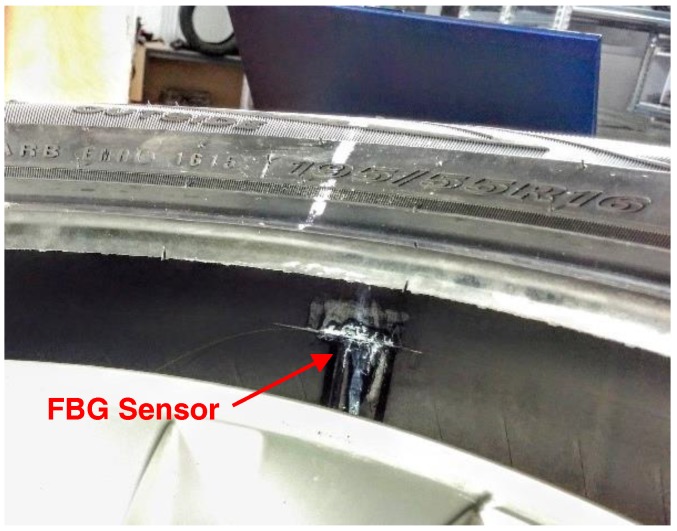
FBG sensor glued on the carcass of the tire.

The FBG sensor is embedded within the tire: the cladding of the naked fiber is glued along the inner side of the carcass, as in Figure 2. The selected adhesive is an epoxy glue with primer for polyethylene and mostly polyolefins and silicone materials: Loctite^®^ 406™ Prism Instant Adhesive and Loctite^®^ SF 770. 

The interrogator is responsible for both light source and spectrum analyzer: a tunable laser fires a selected infrared wavelength light into the fiber array. An optical circulator is placed between the FBG sensor and the light source, which separates the fired beam from the reflected one, being the latter directed to the Optical Spectrum Analyzer (OSA).

The SmartFibers^TM^ SmartScan [39] is the interrogator chosen for this project: the device can have a multiplexed array up to 16 FBGs per channel, with up to four independent channels, in a 40 nm wide bandwidth. The scan frequency is 2.5 kHz in full bandwidth mode (1528–1568 nm), 25 kHz reducing the scan bandwidth. The case dimensions are 140 mm × 110 mm × 70 mm and the weight is 0.9 kg. The information collected from the interrogator is sent via Ethernet to the workstation, on which a suitable software is installed. This allows a remote-lab as the interrogator and the measuring device can be far away from the workstation. 

The SmartSoft software is the frontend interface, where the user customizes the measurement parameters, i.e., number of sensors, sample rate, data averaging, gain application, distance compensation, etc., and permits data log. 

**Figure 3:**
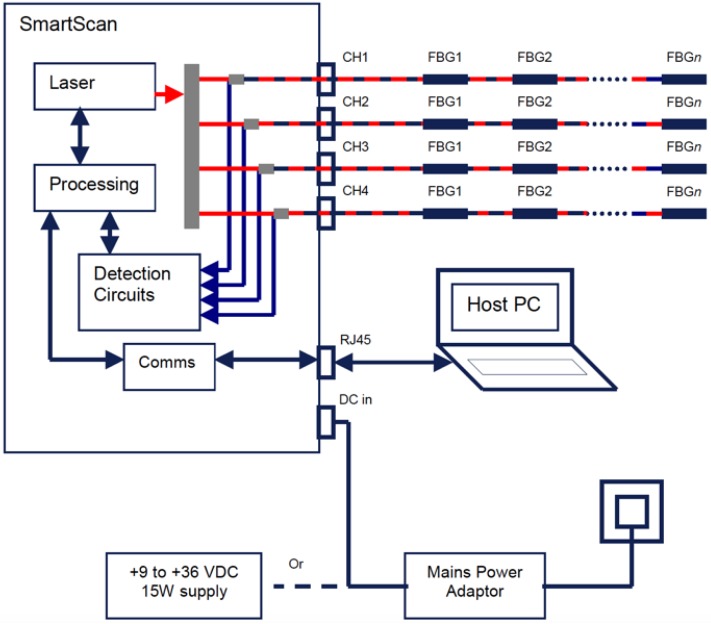
Interrogator logic scheme.

A sketch of the optical sensing chain is provided in Figure 3. 

FBG sensors allow distributed sensing over significant area by multiplexing up to 16 sensors within each fiber and up to 64 sensors total. In the prototype system presented in this paper, only one optical line with a single FBG sensor equips the measurement device, whose length is 10 mm and λ_b_ = 1543 nm. 

This choice is also motivated by the fact that the installation of a single sensor avoids the spectra shouldering, which may arise when two or more sensors are multiplexed and large deformations are involved. Given the low elastic modulus of the rubber in comparison with the silica, the bandwidth available for each FBG sensor needs to be as large as possible. 

**Figure 4:**
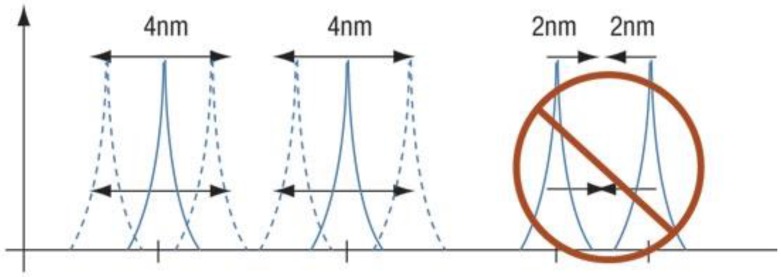
FBG sensors overlapping phenomenon overview.

Namely, let us consider two FBG sensors on the same optical line, whose bandwidths are contiguous, as in Figure 4. When the sensors are stretched, the original peaks in their respective spectrum (solid lines) are displaced (dotted lines), causing a potential overlap of the two spectra. In this case, the wavelength analyzer is no longer capable to separate the two single contributions, producing data loss from both sensors in the worst case.

The maximum measurement allowed by the interrogator for a sensor with $\lambda_{B}=1543$ nm, in pure mechanical strain, is obtained from Equation (3) as follows:

(3) $$\varepsilon_{\text{min/max}}=K_{\varepsilon }\frac{\Delta \lambda_{\text{min/max}}}{\lambda_{B}}$$

where $K_{\varepsilon }$ is a constant of proportionality (about 1.27 × 10^6^ με), $\Delta \lambda_{\min}$ is 15 nm and $\Delta \lambda_{\max}$ is 25 nm. The calculated measurement range is [–7400 με, +20577 με]. The stress limit of the optical fiber is about 0.7 GPa, which, with a Young’s modulus of 16.56 GPa [40,41,42] produces a maximum strain range of about 42300 με to guarantee the fiber integrity. This range lies within the limits of the interrogator.

### 2.2. The Device for the Optical Measurement

**Figure 5:**
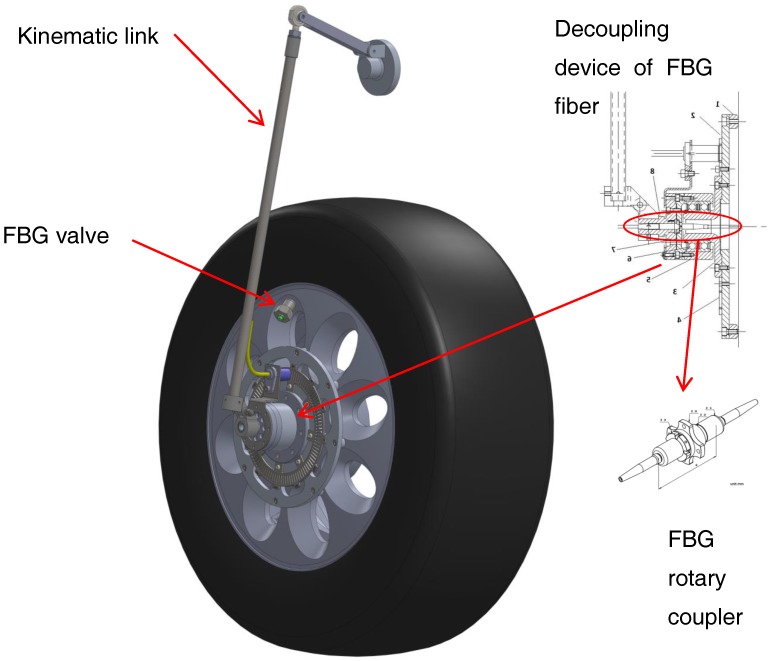
Computer Aided Design-CAD of the wheel with in evidence the FBG valve, the FBG rotary coupler and the kinematic link.

Two main problems are involved in the realization of the present smart tire device: (i) the need to guarantee the pressure seal avoiding any air leakage, still permitting the optical fiber to come out from the inner of the tire, and (ii) the need of decoupling the circular motion of the FBG line within the tire, from the part of the optical line connected to the interrogator, which is indeed integrated to the car body. The general view of the experimental device approaching the solution to problems (i) and (ii) is graphically presented in Figure 5. One can identify: the body of the valve, the support case of the optical joint, and the kinematic linkages needed to carry the optical line on board of the car. 

**Figure 6:**
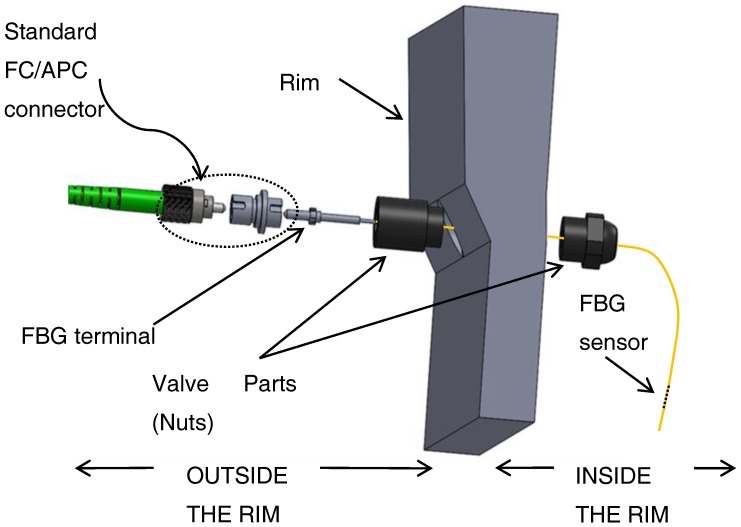
Exploded diagram of FBG valve.

The FBG sensor within the carcass is connected to the optical line, and this is carried outside the tire by a hole drilled into the rim. A specific feedthrough device has been designed, similar to a sealing valve, which is composed by a series of nuts drilled along their symmetry axis, and mounted on the rim as shown in Figure 6. 

After the insertion of the fiber, the internal channel of the feedthrough valve is sealed with silicon to prevent the optical fiber from abrupt bending. The presence of this polymer also inhibits possible air leakages. 

**Figure 7:**
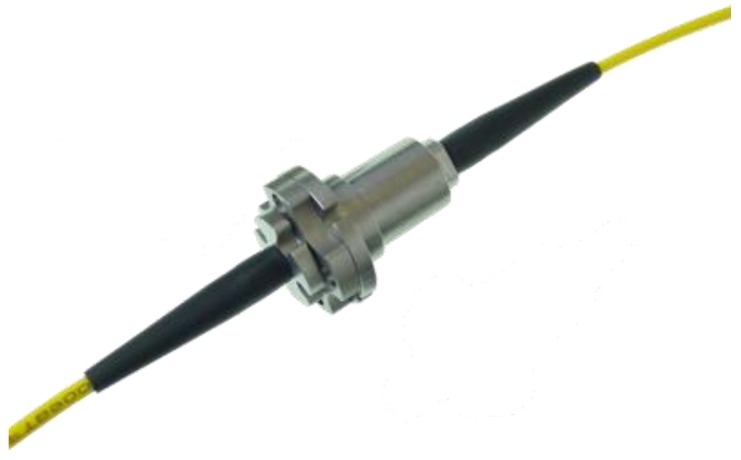
Picture of the optical rotary coupler.

An optical rotary coupler [43] was adopted to couple the rotating part of the fiber to the fixed one. This coupler was mounted within a purpose-designed specific support case. Figure 7 shows a picture of the joint. The trick consists in using a pair of lenses that transmit the light beam between the two separated optical lines, without any mechanical contact between the two fiber ends. The two lenses are indeed each integrated with two cylindrical elements, respectively, that are parts of a mechanical journal bearing. Namely, one of the cylinder is integrated with the car chassis, the other with the wheel. This optical joint can turn at a maximum angular speed about 2000 rpm. Its dimensions and weight are very small, with the maximum diameter about 1 cm, and the weight about 10 g. The optical terminals of the rotary coupler are connected to the optical line coming out from the tire, and the optical line towards to the interrogator, respectively. In the mounting scheme adopted, the axis of the optical coupler must be aligned with the wheel axis (see cross-section in Figure 5). 

**Figure 8:**
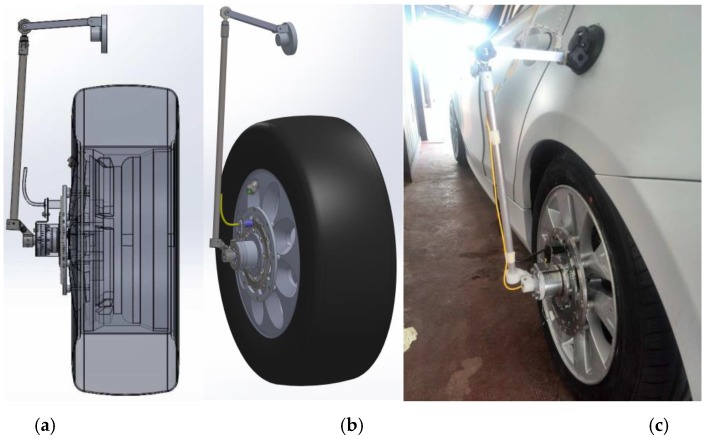
Support case onboard installation ((**a**) CAD model; (**b**) perspective; (**c**) actual prototype).

The third element of the device, represented in Figure 5, is a 3D kinematic chain linkage (articulate quadrilateral with ball joints) that follows the vertical translation of the wheel, while impeding the rotation of the second lens of the optical joint. When mounted on the steering front wheel, the kinematic linkages also allow for the wheel rotation about the vertical axis. A rendering and the final device mounted on the car is shown in Figure 8.

### 2.3. Phonic Wheel, Accelerometer and Other Sensors

The Optyre project employs other sensors besides FBG, which are connected to a data acquisition hardware that is the LMS Scadas Mobile [44]. The measurement system is connected to a host workstation through an Ethernet connection.

The additional sensors chain is composed by: (i) a magnetic Hall effect sensor for the angle and angular speed detection, (ii) a uniaxial accelerometer for the measurement of the vertical acceleration of the wheel axis, (iii) a dynamic temperature sensor for the detection of the thread temperature, and (iv) a GPS system for the global position detection and for the timing reference of the vehicle on which the hardware is installed.

**Figure 9:**
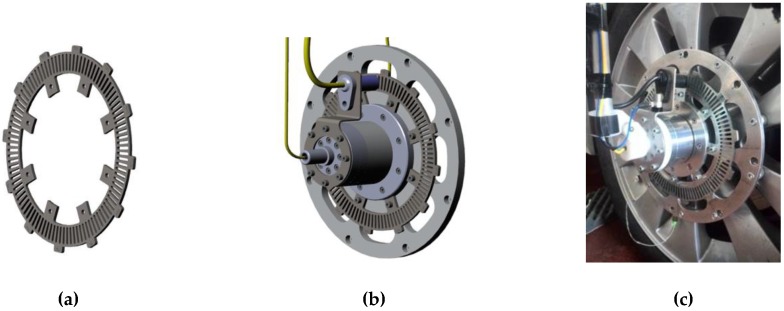
Phonic wheel design: in (**a**) the perspectives; in (**b**) the assembly; and (**c**) a picture of the phonic wheel installed on the support case.

A few sensors, such as the tachymetric logging system and the accelerometer, are linked to the support case, as shown in Figure 9. 

The tachymetric measurement system is composed by a phonic wheel and a magnetic Hall effect sensor. The phonic wheel is obtained from a galvanized ferritic steel disk with an alternation of 100 vacuum and 100 bulk sections for the whole turn, recreating a 1.8° spatial grating, as in Figure 9.

The Hall effect sensor selected for the project is the sensor model AA020020 [45] by Elen srl, which has a maximum acquisition frequency of 15 kHz, corresponding to a maximum detectable speed of 148 km/h, given the actual dimension of the phonic wheel and considering at least three data samples in each vacuum or bulk section.

The Hall effect sensor and the phonic wheel are mounted on the support case, on the stator and on the rotor, respectively, as shown in Figure 9.

The accelerometer is a PCB^®^ axial ICP^®^ accelerometer (Integrated Circuit Piezoelectric 352C33) with acquisition within the range [0.5 Hz, 10 kHz], measurement range ±50 g and sensitivity of 100 mV/g. This sensor is installed on the stator side of the case, to detect the z-axis acceleration of the wheel. 

The data acquisition hardware is provided with a GPS system, to record the historical movements of the car and to synchronize different acquisition systems, by the adoption of a global clock. The installed GPS records longitude, latitude, altitude, global and uniaxial speed and the number of achieved satellites. The antenna of the GPS has been installed on-board.

An optical temperature sensor has been installed to monitor the thread temperature. The sensor is the Optris CSmicro [46] LT-15 pyrometer, which has a measurement range between 0 and 350 °C, and a response time of 30 ms. This sensor is connected to the Scadas. The sensitivity is 10 mV/°C. The sensor has been installed on the vehicle, and the detection spot has an area of 1 cm^2^ of the tire thread in the proximity of the footprint side. The temperature is measured in the neighborhood of the leading edge of the contact patch, in order to detect the temperature while the FBG sensor is closer to the contact area, and to preserve sensor life: whenever the sensor was placed in front of the trailing edge of the tire, it would be exposed to debris.

**Figure 10:**
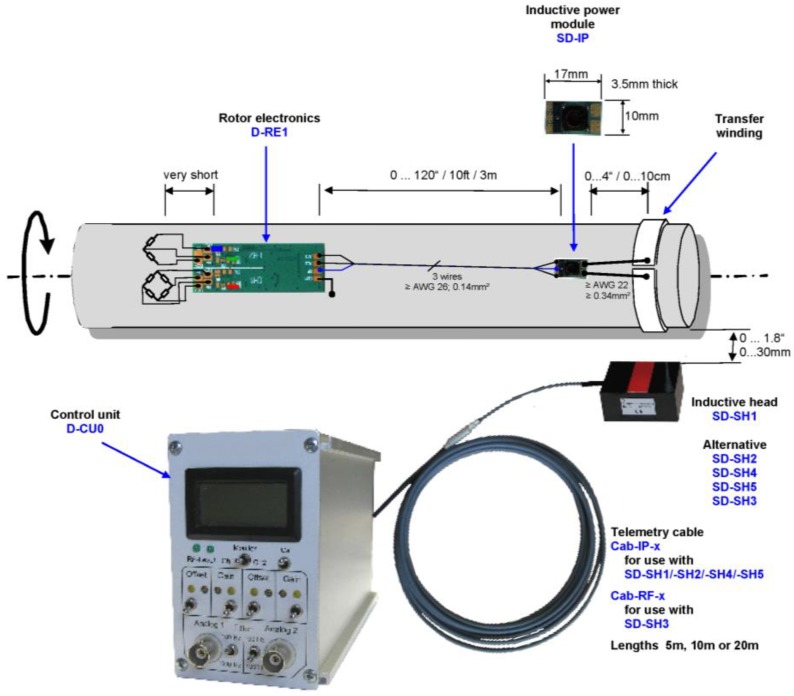
Radio Frequency data transmission system, from PCB^®^ datasheet [47].

The smart tire will be equipped in the near future with a system [47] for the detection of the drive-wheel torque, which measures the strain of the rotating semi-axle, and then, according to Hoffmann [48], it provides the measurement of the torque. The measure is carried out with the use of a radio-frequency module to guarantee an easy data transmission. This supply chain is composed of two main parts, one installed on the axle, and one on the chassis, as in Figure 10. The output of this system is the output voltage of the Wheatstone bridge on which the strain gauges are connected. 

An ICP^®^ sensor for detection of the dynamic pressure will be installed in the inner tube and connected to a parallel branch of the radio-frequency system. The same supply chain described for the torque sensor has been chosen for the data information transmission regarding the dynamic pressure on the internal chamber of the tire. 

**Figure 11:**
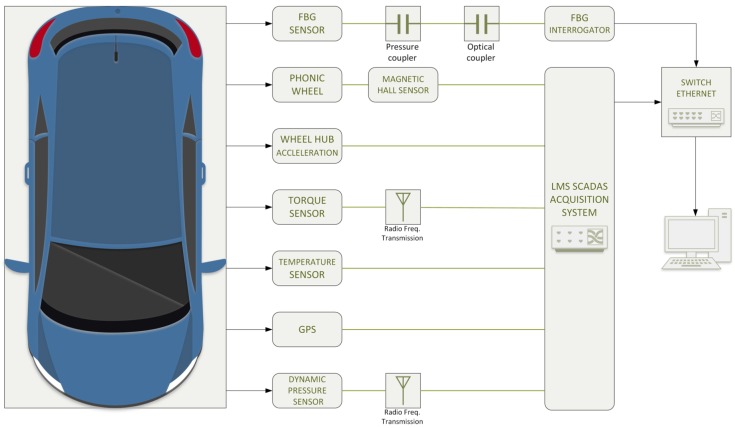
Sensing line conceptual scheme.

Finally, Figure 11 shows an overview of the hardware setup of the project.

The SCADAS data acquisition system can be configured in different ways, and the version used for the project is equipped with 16 VB8 ports for the connection with the sensors. In terms of maximum acquisition frequency, the hardware allows detecting up to 102 kHz, splittable in three different ranges, i.e., Static, Vibrational, and Acoustic, according to the nature of the inspected phenomenon, while the GPS refreshes the data at a frequency about 4 Hz and filtered at 1 Hz.

Actually, the Optyre project provides an alternative solution to miniaturize the hardware. The authors are designing a single compact device containing all the measurement chain, all in one. The new device will exchange data through wireless connections and it will be supplied directly inside the tire by a solution based on the study of the waveguides generated in the tire [49], in order to realize micro-scale devices that exploit the energy pumping phenomenon, as presented in [50], to harvest and collect energy from vibrations and wave-propagation phenomena.

## 3. Tuning of the Acquisition Hardware

The experimental campaign begins with the validation of the measurement chain. The phonic wheel presents some irregularities due to the tolerances of manufacturing process and to the assembly, causing a not negligible error in the angle span of the single element, therefore a calibration of the tachymeter is required. 

**Figure 12:**
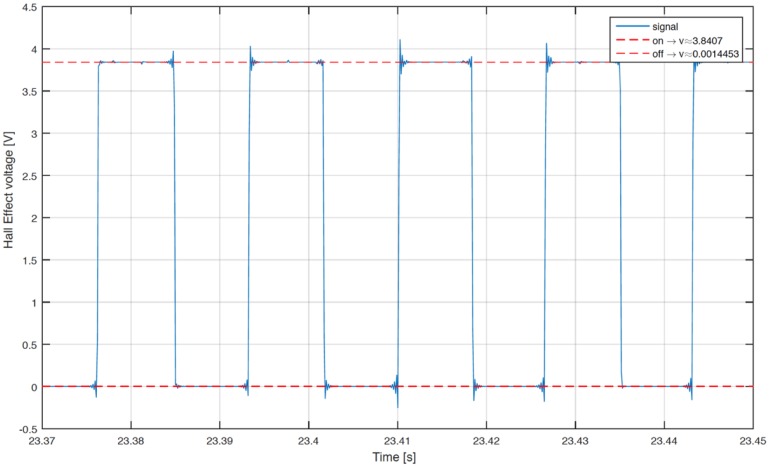
Sample of raw data from the Hall effect sensor.

**Figure 13:**
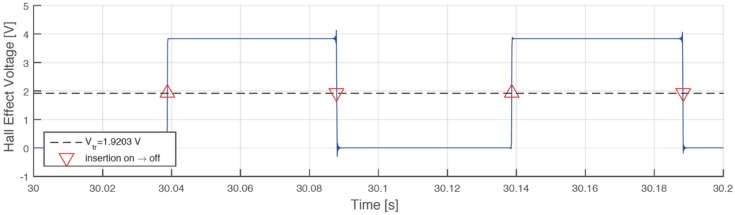
Bulk/void shift identification.

**Figure 14:**
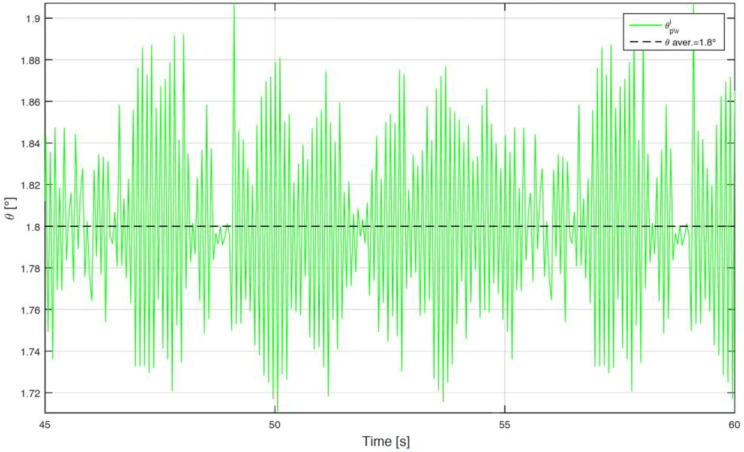
Bulk/void angular dispersion.

**Figure 15:**
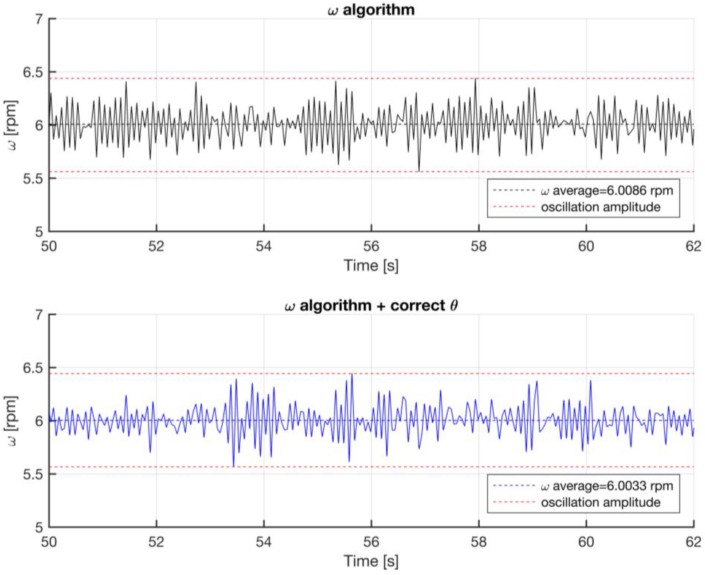
Angular speed correction algorithm results: raw angular speed vs correct angular speed.

A correction algorithm has been built up to identify the phase shift between bulks and voids. In the matter in question, the tachymetric system has been put on a constant speed rotating system, identifying the morphology of every full or empty element, with the aim of calculating the correct phase shift towards a least squares technique. Figure 12 shows the raw voltage output from the Hall effect sensor. Starting from the data, the algorithm identifies the time instant in which there is the passage between bulk and void or vice versa, as shown in Figure 13. The angular distribution of the elements is plotted for a single turn in Figure 14, where the angular dimension of the elements swings between 1.7° and 1.9° with an average value of 1.8°. After the statistical evaluation of the angular dimensions of the single element, it is possible to evaluate the angular correction of each sector. The results of the correction algorithm are shown in Figure 15, where it is possible to notice the benefits of the correction algorithm on the measure of the angular speed. 

**Figure 16:**
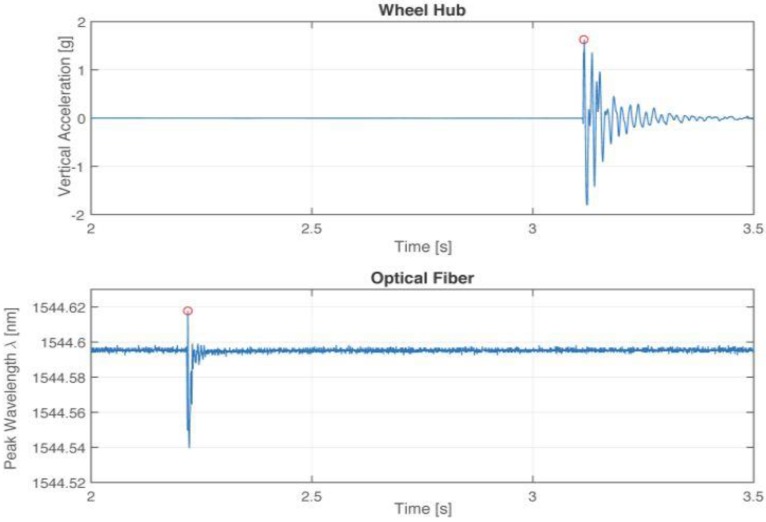
Raw data appearance before synchronization: (**top**) the time history of the vertical acceleration, in g, of the wheel hub, and (**bottom**) the time history of the peak wavelength $\lambda_{B}$ of the FBG sensor, in nm.

It should be noted that the output of the interrogator cannot be connected as an input to the SCADAS system because of the digital nature of the data transmission system of the interrogator. Therefore, to obtain comparable data from the different acquisition hardware, the two devices need to be synchronized. This task was manually carried out by the operator at the stage of the project, causing a small time delay between the two sets of data logging: one caused by the reaction time of the operators in charge for the data recording, the other cause is the delay of the two different software packages on starting the data logging. The proposed solution is the stimulation of the system with a recognizable perturbation, such as a hammer hit on the tire with the tire wheel at rest. The time histories of the two data sets are shown in Figure 16, where the red circles show the time instants after which the hammer hit takes place in respect to the origin of the time axes. A developed algorithm provides the proper shift of the time axes, so that the hammer hit is the zero time for both time axes.

## 4. Experimental Campaign

The described measurement device permits to observe for the first time which are the real elastic phenomena that take place inside the tire in real operating rolling conditions.

All the previously described components of the measurement chain are used to correlate the circumferential strain acquired by FBG sensors, directly to the tire temperature and pressure, wheel vertical displacement, angular position and speed of the tire. 

Two types of measurements characterize the experimental campaign: one performed with the vehicle in steady state motion, and the other involves accelerated/decelerated motion: both are carried out along a rectilinear trajectory and real roads, to keep into account, in this phase of the analysis, only forces and torques involved with the longitudinal motion of the car. 

**Figure 17:**
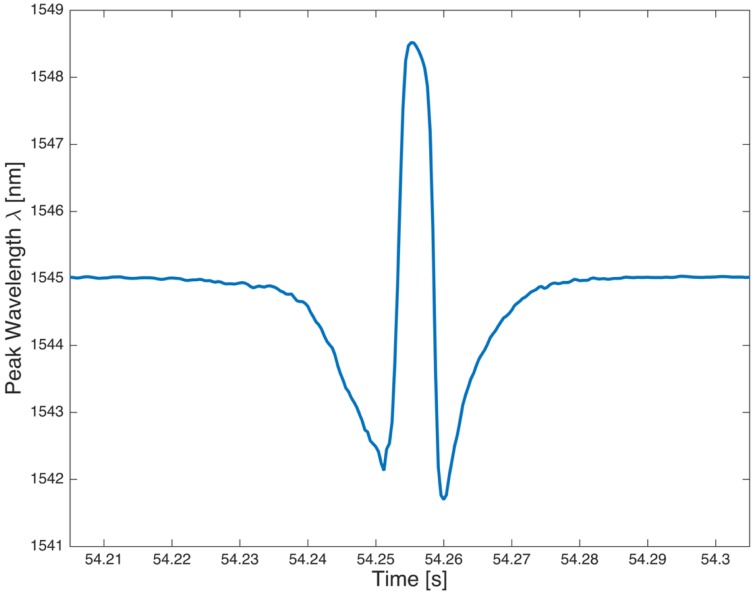
Reflected wavelength for a complete wheel turn.

Figure 17 shows the time history of the wavelength changes acquired by FBG sensor, during one single revolution of the tire. The y-axis is directly proportional to the circumferential strain of the tire, as shown by Equation (2). As far as the sensor is roughly in correspondence of the center of the contact patch, the strain achieves the maximum value, otherwise, when the sensor is far from the contact patch the strain remains close to zero and the strain values are main influenced by temperature and inflation pressure.

**Figure 18:**
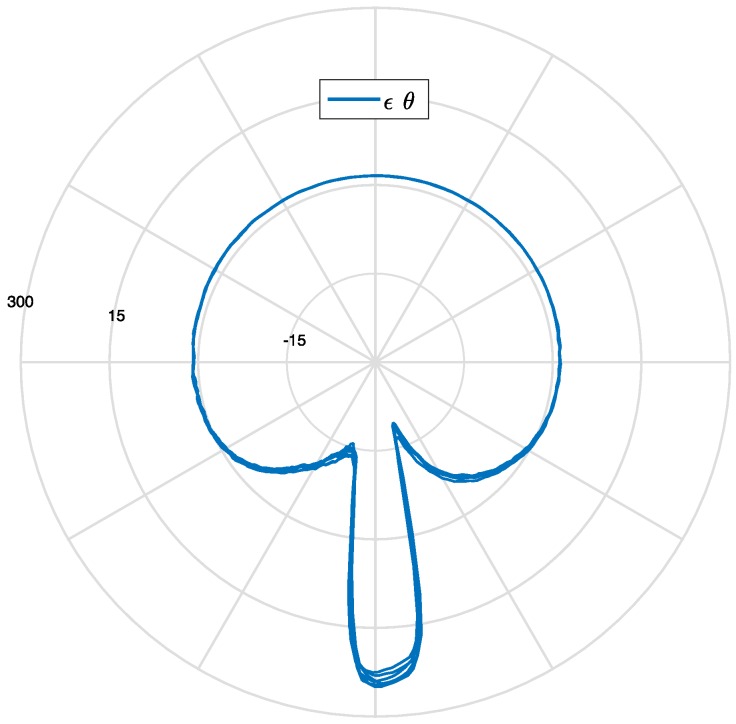
Polar plot of circumferential strain (μ strain) as function of the wheel rotation angle (θ ).

**Figure 19:**
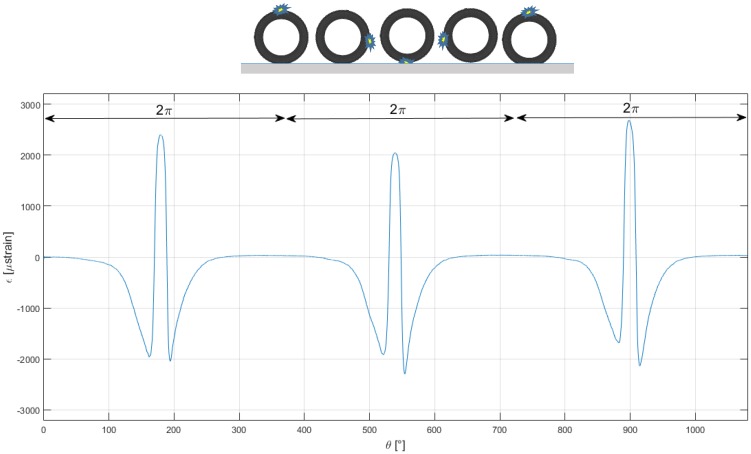
Plot of strain and FBG sensor position as function of the wheel rotation.

Figure 18 shows the polar plot of the circumferential strain after several tire rotations in a steady state motion: curves belonging to different rotations mostly coincide. Figure 19 summarizes how the measured tire strain is correlated to tire angular position, detected by the phonic wheel system.

**Figure 20:**
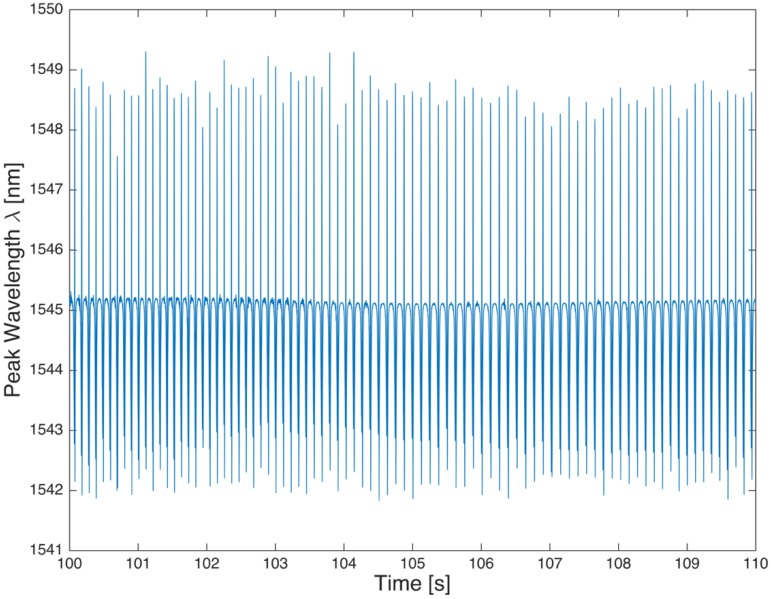
Strain log for the vehicle in steady motion at 40 km/h.

**Figure 21:**
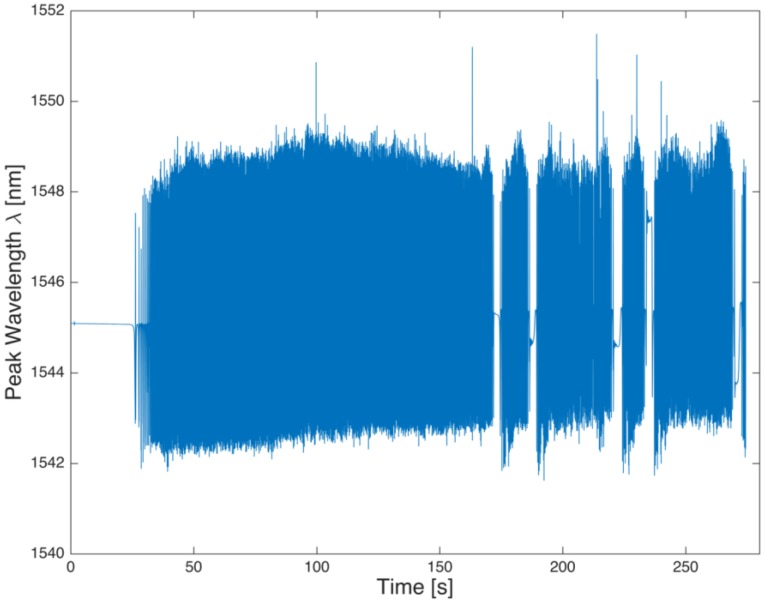
FBG wavelength log for a random speed straight run.

**Figure 22:**
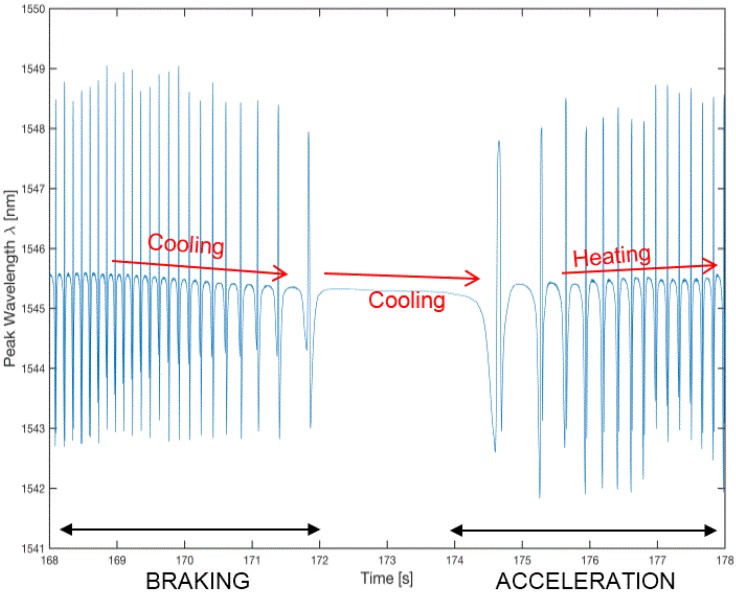
Detail of Figure 21 between 168 s and 178 s with a stop-start sequence between 172 s and 174.5 s.

Figure 20 shows the strain detected by the FBG sensor in a 10 s steady state motion at 40 km/h, Figure 21 shows an accelerated motion, and in Figure 22 shows a magnification of Figure 21 taken around 163.5 and 182.5 s, which shows a brief stop followed by a resumption of the motion. Figure 22 shows the cooling of the tire during the stop of the vehicle, as indicated by the decreasing trend of the signal, according to the second addendum in Equation (2). 

**Figure 23:**
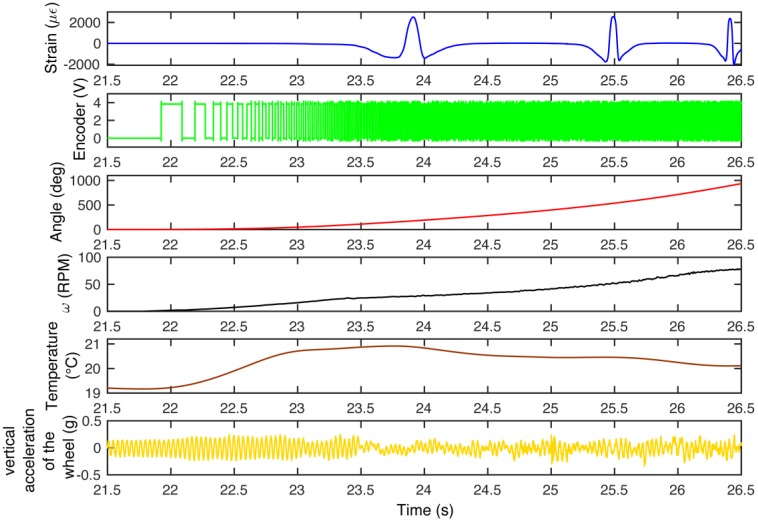
Multi-sensor data logs: Strain, Encoder output voltage, Angle, Rotational speed, Temperature, and Wheel hub vertical acceleration, respectively.

**Figure 24:**
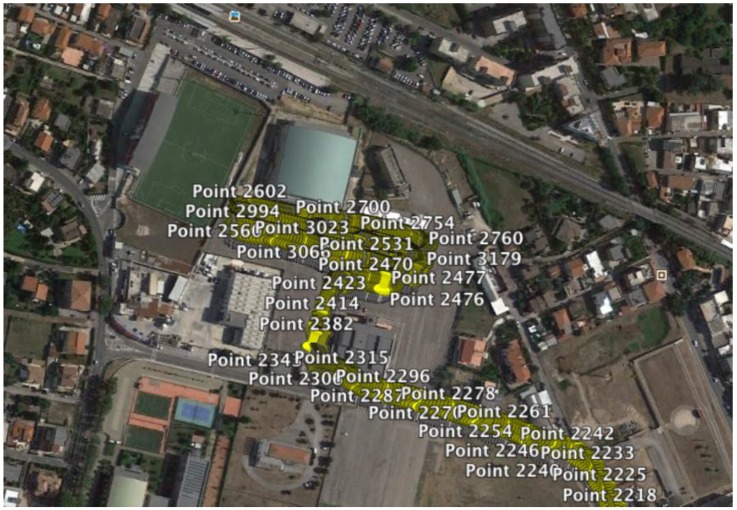
GPS tracking data log.

Figure 23 shows a five seconds window that summarizes the recorded data from all the sensors between 21.5 s and 26.5 s for the accelerated/decelerated motion. The data show an acceleration from 0 km/h to approximately 8.5 km/h, inter alia, the motion starts at about 22 s, where the encoder signal, shown in the second row of the picture, switch the square wave, caused by the approaching of the Hall effect sensor from a vacuum to a bulk section. The deformation of the FBG sensor is represented in the first row. In the picture it is possible to recognize up to three passages of the sensor in the footprint. The third row shows the angle increase due to the start of the motion, while the fourth row shows the angular speed of the wheel; from the second to the fourth row the data are collected from the phonic wheel system. The graph representing the temperature shows that at the beginning of the motion the temperature initially increases, after which the temperature stabilizes to a new value. The last row shows the vertical acceleration of the wheel that can be directly associated with the vertical load acting on the wheel, and then deforms the contact patch. Furthermore, it is possible to follow the vehicle using a Global Position System. The Figure 24 shows a picture of the geo-referenced data, in terms of latitude and longitude, plotted with the satellite image in the background. It is possible to identify the area in the background of the picture: the Vehicle Dynamics and Mechatronics Laboratory of Sapienza, University in Rome, located in Cisterna di Latina, Latina, Italy.

## 5. Reconstruction of Fundamental Tire Phenomena

The data acquired with the built-in hardware is processed with algorithms developed in related works [33,34,35], for the reconstruction of the phenomena at the contact patch, such as the angular position of the maximum detected deformation, the footprint length, the leading and trailing edges, the transition abscissa between stick and slip region and the evaluation of the rolling resistance.

**Figure 25:**
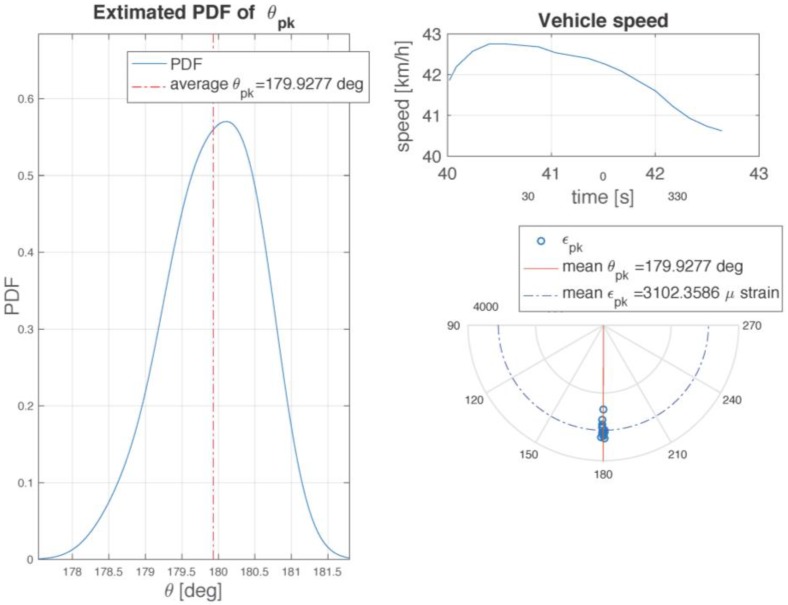
PDF and polar plot of strain peak angular position and plot of local speed over time.

**Figure 26:**
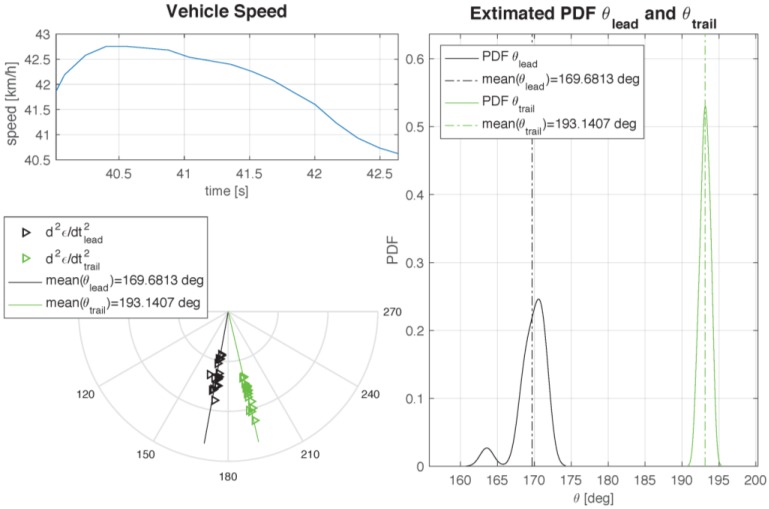
PDF and polar plot of footprint extrema and plot of local speed over time.

An important parameter for the study of the phenomena at the contact patch is the angular position $\theta_{max}$ at which the maximum circumferential deformation within the tire is reached, as shown in Figure 25, for a few rotations in constant speed motion. For each tire revolution, the peak of the circumferential deformation is detected together with the associated angular position $\theta_{max\;i}$. The polar plots of the angular positioning of each detected $\theta_{max\;i}$ are reported in Figure 26. This distribution of points permits to derive a probability density function-PDF $pdf\left(\theta_{max}\right)$, which is represented in Figure 25. The PDF shows that both the average and the maximum value of the curve lie in the neighborhood of 180 degrees, but introducing a clear displacement of the pdf peak in the direction of motion of the wheel center, making the distribution asymmetrical. This effect amounts to the unavoidable presence of dissipation effects that perturb the static symmetric distribution of the vertical load, producing the well known displacement of the vertical force application point towards the direction of the wheel center [51]. 

The same statistical approach also suggests the estimation of the footprint length. According to [33], the leading and the trailing edges, defining the initial and final points of the footprint, correspond to the maxima of the second time derivative of the deformation: in analytical terms, the deformation speed rate exhibits stationary points at the two edges. The polar plot shows the positions of these two points (black for the leading edge, green for the trailing edge, respectively) in a set of nominally identical rotations. It is interesting to note that the two sets of points are aligned along two radii that are not symmetrical with respect to the ideal tangent point of the wheel. In fact, the footprint appears to be, once again, displaced in the direction of the wheel center speed, as a consequence of the dissipation effects. For each set of points one can build the corresponding PDF. Figure 26 shows both the PDFs, determined at a roughly constant wheel speed. The best estimate of the footprint length is the distance between the peaks of the two PDFs curves, that amounts to the distance between the average positions of the trailing and leading edge points. 

**Figure 27:**
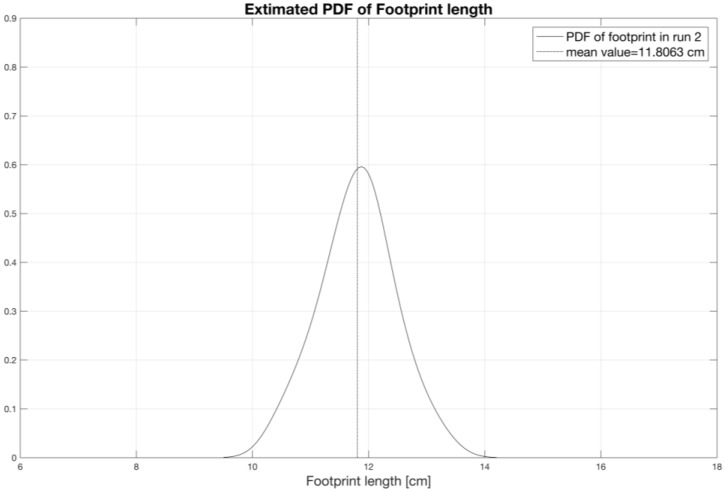
Contact length PDF in two different tests.

Using the same statistics, Figure 27 shows the PDF curve of the footprint length, whose average length, for the analyzed case, is about 12 cm. 

**Figure 28:**
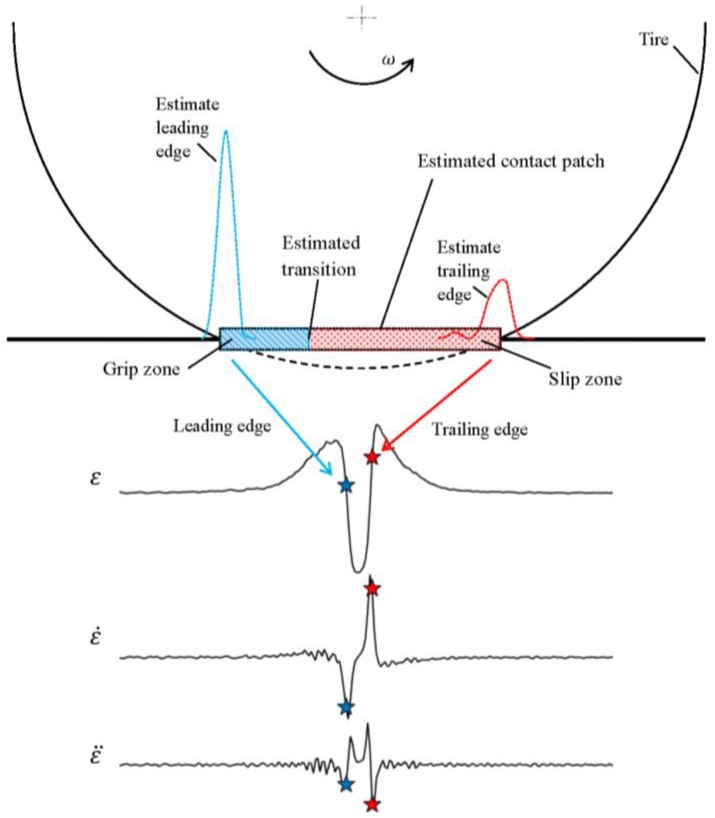
Summary of the contact patch, transition abscissa and grip/slip area identification.

The study of the grip phenomena is also possible, and generally very difficult at the actual state of the art. Following the analyses presented in [33,34], it is possible to identify the transition region between grip and slip areas using special algorithms. With the present measurements, we rely only on the detection based on an abrupt variation on the time history of the first time derivative of the strain [33,34]. Figure 28 shows the strain, its first time derivative, and the identified transition abscissa. 

A parameter that is strictly connected to the transition abscissa is the slip ratio. This parameter is calculated dividing the value of the footprint surface in slip conditions for the value of the whole footprint surface. For small slip ratios, i.e., in the case of light torque applied, the transition abscissa falls in the neighborhood of the trailing edge, as presented in [33], and the slope change along the strain curve may become hardly perceptible. In these cases, the experimental identification of the transition abscissa is more problematic, since ambient and numerical noise together with inadequate time sampling may hide the discontinuity on the first derivative of the strain curve. 

The measured data also allow the estimation of the dissipated power due to hysteresis [35], where a hysteresis factor is found that explicitly depends on the square of the deformation speed. 

In the end, the data collected in the project will be combined, in forthcoming activities, with some advanced models presented in [52,53], to develop a model for the tire structural health monitoring and damage detection. 

## 6. Comparative Analysis

The performances of the developed device are here compared to the most relevant solutions in this field, which provide state of the art tools for analyzing the contact patch phenomena in a rolling tire. 

One of the most acknowledged project is The Cyber™ Tyre [30], developed by Pirelli, which uses a tri-axial accelerometer glued onto the internal carcass of the tire, to estimate the tire-road contact dynamics and feed the control logic that governs the active safety of the car. The energy is scavenged with a nonlinear electro-magnetic device [54]. The energy harvested and the raw acceleration data are managed by a DSP—Digital Signal Processor. 

With respect to the Optyre project, the presence of the DSP suggests that (i) the transmission rate and (ii) the sampling frequency of the data are limited by (iii) the power consumption, affecting the quality of the measured data. Moreover, (iv) the space resolution of the acquired signal is limited by the intrinsic dimensions of the accelerometer sensor, and finally (v) a radio transmitter should be embedded into the tire. All these limitations are overcome with the proposed technology.

Another related project is based on image processing [32] and has been developed by the Department of Mechanical Engineering of the University of Science in Tokyo. The system relies on the estimation of vertical and frictional loads at the contact interface and uses a CCD camera that monitors the deformation of the internal tire carcass, on which a series of rubber blocks are attached accordingly to a specific pattern. 

As in the Optyre project, this is an embedded wireless device for the strain measurement, it has the advantage of using a contactless sensor, at the cost of (i) the invasive dimension of the CCD, whose power is supplied by a battery that (ii) needs to be recharged, (iii) substantial modifications of the inner liner caused by the rubber blocks and (iv) a radio receiver needs to be installed. Given the spacing of the rubber blocks and the CCD resolution, (v) the spatial accuracy obtained here is much lower than in Optyre. 

The same authors also developed a strain monitoring device using a wireless system, through the embedding of a RC oscillating circuit in the inner tire [26]. A portion of the steel wire belt of the tire is the capacitor, and its deformation induces a change in the electrical capacity, which wirelessly permits to identify the tire strain, thanks to a change in the frequency response of the oscillating circuit. With respect to the Optyre technology a clear advantage of this device is to use the carcass of tire as a sensor, nevertheless with this solution (i) the measurement is averaged on a large tire surface, that is not negligible. Moreover, (ii) the frequency bandwidth of the oscillating circuit limits the maximum operating speed, finally, (iii) road tests are not reported. 

The CAIS-Contact Area Identification System [31], developed by Bridgestone, is able to estimate the contact patch, the load and side force, thanks to direct measurements of strain, acceleration, pressure and temperature, with a wireless sensor placed inside the tire. The system can also classify the road profile and estimate the tire tread wear. This solution may allow real-time testing but, as mentioned in [31], (i) an energy harvester is needed and not designed yet. The data processing algorithms, on which this technology is based, rely on the assumption that the highest speed of deformation occurs when the monitored points reach the edges of the contact patch. About this hypothesis, the authors showed in [33] that using the formulas presented in [31], the contact patch is underestimated. It was indeed demonstrated, theoretically and experimentally, that (ii) the extrema of the deformation speed fall inside the contact patch [33].

In conclusion, although Optyre presents some important advantages with respect to aforementioned technologies, such as the direct strain measurement with negligible insertion errors, a high spatial resolution, a multiple points measurement within a single fiber, high strain sensitivity, high frequency measurements and the absence of any sort of power source within tire, it is still a prototypal device that needs to be engineered for a widespread commercial use.

## 7. Conclusions

The experimental apparatus developed in the Optyre project has been presented in this paper. The innovative experimental system relies on the embedding of optical lines with FBG sensors within the tire carcass, together with the use of other sensors, such as a phonic wheel, a uniaxial accelerometer, a dynamic temperature sensor and a GPS, which allow the real-time identification of key parameters, such as the tire stress during rolling, the residual grip and the rolling resistance, as shown in previous papers and will be presented in forthcoming publications, which will be focused on both the development and the engineering of a vehicle dynamics control system based on the presented hardware. 

The validity of the developed smart tire was investigated with an extensive experimental campaign, and by comparison with other existing technologies. Results confirm the potential of the present apparatus, which provides signals with high resolution and accuracy, does not require energy harvesting or complex transmission systems, and is capable of long-term acquisitions under adverse conditions.

The reliability of the experimental setup developed in the Optyre project suggests moving towards new research directions, in order to investigate further phenomena involving the tire revolution: the study of the impact dynamics of the leading edge applying the model presented in [55]; the evaluation of the tire-road dynamics in presence of interstitial fluids, such as in aquaplaning conditions, following the thesis developed in [56]; the development of a new high order constitutive relationship between stress and strain, for the complex structures of the tire, through models close to the ones suggested in [57]; or the use of the theory presented in [58] to evaluate a richer phenomenology of the contact between rough surfaces.
